# Expression of epithelial-mesenchymal transition-related markers and phenotypes during breast cancer progression

**DOI:** 10.1007/s10549-020-05627-0

**Published:** 2020-04-16

**Authors:** Charlotte Levin Tykjær Jørgensen, Carina Forsare, Pär-Ola Bendahl, Anna-Karin Falck, Mårten Fernö, Kristina Lövgren, Kristina Aaltonen, Lisa Rydén

**Affiliations:** 1 Division of Oncology and Pathology, Department of Clinical Sciences, Lund, Lund University, Medicon Village, Building 404, 22381 Lund, Sweden; 2 Department of Surgery, Helsingborg Hospital, Helsingborg, Sweden; 3 Division of Translational Cancer Research, Department of Laboratory Medicine, Lund University, Lund, Sweden; 4 Division of Surgery, Department of Clinical Sciences, Lund, Lund University, Lund, Sweden; 5 Department of Surgery, Skåne University Hospital, Lund, Sweden

**Keywords:** Breast cancer, Tumor progression, Primary tumor, Recurrence, Epithelial-mesenchymal transition, EMT phenotypes

## Abstract

**Purpose:**

The study aimed to investigate expression of epithelial-to-mesenchymal transition (EMT)-related proteins and phenotypes during breast cancer progression and to relate this to patient outcome.

**Methods:**

Protein expression patterns of E-cadherin, N-cadherin, twist, and vimentin were examined by immunohistochemistry on formalin-fixed paraffin-embedded samples from primary tumors (PTs) (*n* = 419), synchronous lymph node metastases (LNMs) (*n* = 131) and recurrences (*n* = 34) from patients included in an observational prospective primary breast cancer study. Markers were evaluated individually and combined as defined EMT phenotypes (epithelial, mesenchymal, partial EMT, and negative). EMT profiles were compared between matched tumor progression stages, and related to clinicopathological data and distant recurrence-free interval (DRFi).

**Results:**

N-cadherin-positivity, vimentin-positivity, mesenchymal and partial EMT phenotypes were associated with more aggressive tumor characteristics such as triple-negative subtype. Single EMT markers and phenotype discordance rates between paired tumor samples were observed in the range of 2–35%. Non-epithelial phenotypes were more frequently identified in recurrences compared to PTs, however, no skewness of expression or phenotype was detected between PTs and matched LNMs or between PTs and matched recurrences (Exact McNemar test). Interestingly, patients with a twist positive PT had shorter DRFi, compared to patients with a twist negative PT (hazard ratio (HR) 2.4, 95% confidence interval (CI) 1.2–5.1, *P* = 0.02). Essentially, the same effect was seen in multivariable analysis (HR 2.5, 95% CI 0.97–6.6, *P* = 0.06).

**Conclusion:**

The epithelial phenotype was indicated to be lost between PTs and recurrences as a reflection of tumor progression. Twist status of the PT was related to long-term prognosis warranting further investigation in larger cohorts.

**Electronic supplementary material:**

The online version of this article (10.1007/s10549-020-05627-0) contains supplementary material, which is available to authorized users.

## Introduction

Even though the prognosis of breast cancer has greatly improved over the past decades [1], approximately 30% of patients diagnosed with primary breast cancer will develop recurrent disease within 10 years [2–4]. In these patients, metastatic disease is the primary reason for shorter survival, yet the process of metastasis is still poorly understood [5]. Epithelial-to-mesenchymal transition (EMT) is an evolutionarily conserved program active during physiological processes such as embryogenesis and branching morphogenesis of the mammary gland, though also a process reactivated during the pathogenesis of tumor progression and metastasis of carcinomas [5–9]. During this transition, the epithelial cells downregulate epithelial markers, lose features such as polarity and intercellular adhesion, and upregulate mesenchymal markers to acquire features such as a fibroblast-like morphology and increased motility [5–9]. EMT is a reversible process and mesenchymal-to-epithelial transition, the process of regaining epithelial properties, is considered essential for the establishment and outgrowth of cancer cells at secondary sites [5–11]. Recently, tumor cells co-expressing epithelial and mesenchymal markers have been identified, and described as “partial EMT”, and thus represents a continuous spectrum of intermediate states between the epithelial and mesenchymal phenotype [12–16]. Tumor cells with an intermediate state of EMT have been associated with greater metastatic potential and a worse prognosis in breast cancer [12, 13, 16]. The processes of EMT are well recognized in preclinical studies, however, direct evidence of EMT in clinical samples is still lacking [17–21].

Currently, there are no standard biomarkers to demonstrate EMT, however, downregulation of epithelial markers, such as the cell surface protein E-cadherin and the cytoskeleton protein cytokeratin, are considered hallmarks of EMT. Common mesenchymal markers that are upregulated during EMT are cell surface protein N-cadherin, cytoskeleton protein vimentin and the transcription factor twist [22–24]*.* The primary aim of the present study was to evaluate the expression of these five selected EMT-related markers by immunohistochemistry (IHC) in breast cancer specimen to investigate if changes occur during tumor progression by examining paired samples of primary tumors (PTs) and synchronous lymph node metastases (LNMs), and paired samples of PTs and recurrences. Expression of the EMT-related markers were assessed individually and EMT phenotypes were defined based upon the combined expression pattern of the markers to explore the wide spectrum of EMT phenotypes previously reported including partial EMT. We hypothesized that the expression of EMT-related markers and phenotypes will be significantly different between tumor progression stages. To evaluate the clinical significance of EMT-related markers and phenotypes, correlation with clinicopathological factors and patient outcome was analyzed.

## Materials and methods

### Patients

The present study is based on a subset of breast cancer patients previously included in a prospective observational study originally evaluating the presence and prognostic value of disseminated tumor cells in bone marrow [25]. A total of 569 primary breast cancer patients diagnosed between 1999 and 2003 were included (South Swedish Health Care Region: Lund, Landskrona, Helsingborg), and the study will in the following be referred to as the Bone Marrow Metastases (BMM) cohort [25]. The study was approved by the Lund University ethics committee (LU699-09, LU75-02), and all patients included provided written informed consent. Results of the observational study and information about the patient cohort have been described, including biomarker protocols and assessment, in detail previously [25–27]. Patients diagnosed with lobular carcinomas were excluded in the present study, as several studies have demonstrated a different expression pattern of E-cadherin in this type of breast cancer [28–30]. Median follow-up time for patients alive and without distant recurrences was 13.9 years at last follow-up. The latest data on recurrences were retrieved from individual patient charts and causes of death from the Swedish Register of Causes of Death (Central Statistics Office; November 2015).

### Tissue microarray and immunohistochemistry

Formalin-fixed paraffin-embedded archival blocks with tumor tissue from the BMM cohort were retrieved from the archives of pathology departments and one set of tissue microarrays (TMAs) (2 × 1 mm core diameter) was constructed as previously described (Beecher instruments, MD, USA) [26].

Consecutive 3–4 μm sections from each TMA block were cut and transferred to glass slides (Menzel Super frost plus, Thermo Scientific, Germany), dried at room temperature and baked for 2 h at 60 °C in heat chamber. Following deparaffinization and antigen retrieval, IHC staining was performed using Autostainer Plus (Dako Denmark A/S, Glostrup, Denmark). The following antibodies and dilutions were used: E-cadherin (NCH-38, #M3612 Dako Denmark A/S, 1:100), pancytokeratin (AE1/AE3, #3515 Dako Denmark A/S, 1:500), N-cadherin (3B9; #33–39 Invitrogen, 1:25), vimentin (V9, #M0725 Dako Denmark A/S, 1:300) and twist (2C1a, #ab50887 Abcam, 1:10). Counterstain with Mayer’s Haematoxylin was applied for 2 min to each section and a visualization kit K801021-2 (Dako Denmark A/S) was used for all stainings.

Stained TMA sections were scanned (Hamamatsu Photonics, NanoZoomer, software NDP-Scan, Japan), and using the web-based image and data management platform Xplore (Philips) all markers were scored independently by two observers blinded to clinical data [E-cadherin, N-cadherin (KA, CF); pancytokeratin, vimentin, twist (KL, CLTJ)]. Stainings were evaluated for intensity 0–3 (0 = negative, 1 = weak, 2 = intermediate, 3 = strong) and percentage of stained tumor cells. Only invasive tumor cells were assessed and only TMA core biopsies with > 100 invasive tumor cells were included. Samples with differences in assessment between the two investigators were re-evaluated and a consensus decision taken. The highest value of the two cores was used for statistical analysis.

In accordance with similar thresholds used in previous studies, a value of > 10% positive tumor cells independent of intensity was chosen to define positive expression of E-cadherin, pancytokeratin, N-cadherin, vimentin and twist [31–36]. As all samples but one were positive regarding pancytokeratin expression, the majority in the range of 90–100%, this marker was excluded from further analysis. EMT phenotype classification was done based upon the EMT phenotypes proposed previously in breast, small intestinal and esophageal cancer [13, 15, 16, 37, 38]. We stratified all samples where E-cadherin (epithelial marker) and at least one mesenchymal marker (N-cadherin, twist, vimentin) were evaluable into to the following phenotypes of EMT: epithelial type (positive expression of epithelial marker and negative expression of all evaluable mesenchymal markers); mesenchymal type (negative expression of epithelial marker and positive expression of at least one mesenchymal marker); partial EMT type (positive expression of epithelial marker and of at least one mesenchymal marker); and negative type (negative expression for epithelial marker and all evaluable mesenchymal markers).

### Statistical analysis

Markers were analyzed individually and combined as defined EMT phenotypes. The association between EMT-related marker/phenotype expression and different patient and PT characteristics was analyzed with *χ*^2^ test and Fisher’s exact test. Comparison of EMT marker/phenotype status between PTs, synchronous LNMs and recurrences was performed using the exact McNemar test. To evaluate survival effect, Kaplan–Meier survival curves and log rank test was used. Hazard ratios (HR) were calculated by Cox regression and multivariable analyses were adjusted for age, tumor size, lymph node status, Nottingham histologic grade (NHG) and St Gallen breast cancer subtype. Schoenfeld’s test (estat phtest in STATA) was used to check assumptions of proportion hazards and the evidence against proportionality was found to be weak for all markers. Distant recurrence-free interval (DRFi) was chosen as primary endpoint before evaluation of EMT-related markers. DRFi was defined as the time from surgery until verified distant recurrence by radiology/biopsy or breast cancer-related death. For event-free patients, follow-up was censored at last medical follow-up visit. All *P-*values presented are two-sided. No adjustment for multiple testing has been performed. *P*-values should be regarded as level of evidence against the null hypothesis. We follow the advice in Benjamin et al. and use the term suggestive evidence for *P*-values in the range 0.005–0.05 and significant for *P*-values below 0.005 [39]. Statistical calculations were performed using IBM SPSS Statistics (version 24.0, IBM, Armonk, NY, USA) and STATA (version 15.1, StataCorp. College Station, TX, USA). Reporting Recommendations for Tumor Marker Prognostic Studies (REMARK) were followed where applicable [40].

## Results

### Patients and tumor characteristics

**Fig. 1:**
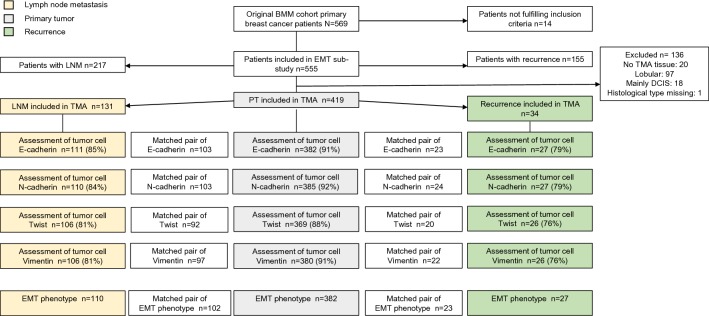
Flowchart of patient cohort and EMT-related marker expression/phenotype in primary tumor, synchronous lymph node metastases and recurrences. Synchronous lymph node metastasis and recurrences were only evaluable in 131 and 34 patients, respectively. Boxes inserted into the flowchart display information of EMT-related marker/phenotype on matched pairs, i.e., numbers of primary tumors and lymph node metastases, and primary tumor and recurrences, respectively. *BMM* bone marrow metastasis, *DCIS* ductal carcinoma in situ, *EMT* epithelial-mesenchymal transition, *N* number, *LNM* lymph node metastasis, *PT* primary tumor, *TMA* tissue microarray

The original BMM trial recruited 569 participants. A total of 14 patients did not fulfill the inclusion criteria [25], thus leaving 555 patients for the present study (Fig. 1). Archival tumor tissue was available in the form of TMA from 535 of the included patients (96%). Excluding samples of histological lobular type, ductal carcinoma in situ and one sample with missing histological status, PT samples from a total of 419 patients (78%), matched synchronous LNMs from 131 patients (24%), and recurrence samples from 34 patients (6%) were included in the final analyses. Patient and PT characteristics of the entire BMM cohort and the subset included in the present study are summarized in Supplementary Table 1 (Online Resource 1). Overall, the characteristics of the 419 included patients were similar to the characteristics of all patients included in the BMM trial.

**Table 1:** Associations between primary tumor epithelial-mesenchymal transition (EMT)-related markers and patient and primary tumor characteristics

Characteristics	E-cadherin +	E-cadherin-	*P*	N-cadherin +	N-cadherin−	*P*	Twist +	Twist−	*P*	Vimentin +	Vimentin−	*P*
	*N *(%)	*N *(%)		*N *(%)	*N *(%)		*N *(%)	*N *(%)		*N *(%)	*N *(%)	
	357 (93)	25 (7)		32 (8)	353 (92)		19 (5)	350 (95)		71 (19)	309 (81)	
Age			0.13^b^			0.37^b^			0.78^a^			0.006^b^
< 50	79 (22)	2 (8)		9 (28)	73 (21)		3 (16)	75 (21)		24 (34)	57 (18)	
≥ 50	278 (78)	23 (92)		23 (72)	280 (79)		16 (84)	275 (79)		47 (66)	252 (82)	
Tumor size			1.00^b^			0.56^b^			1.00^b^			0.02^b^
≤ 20 mm	241 (68)	17 (68)		20 (63)	240 (68)		13 (68)	241 (69)		40 (56)	219 (71)	
> 20 mm	116 (32)	8 (32)		12 (37)	113 (32)		6 (32)	109 (31)		31 (44)	90 (29)	
Nodal status			0.40^b^			1.00^b^			0.63^b^			0.89^b^
Negative	199 (57)	16 (67)		18 (58)	199 (58)		12 (67)	199 (58)		42 (60)	175 (58)	
Positive	149 (43)	8 (33)		13 (42)	145 (42)		6 (33)	142 (42)		28 (40)	125 (42)	
Missing	9	1		1	9		1	9		1	9	
NHG			0.29^b^			< 0.001^b^			0.91^b^			< 0.001^b^
I	89 (25)	3 (12)		3 (9)	89 (25)		4 (21)	87 (25)		10 (14)	82 (27)	
II	153 (43)	14 (56)		9 (28)	160 (46)		8 (42)	151 (43)		20 (29)	144 (46)	
III	113 (32)	8 (32)		20 (62)	102 (29)		7 (37)	110 (32)		40 (57)	82 (27)	
Missing	2	0		0	2		0	2		1	1	
ER			0.35^a^			0.03^a^			1.00^a^			< 0.001^b^
Negative	45 (13)	5 (21)		9 (28)	41 (12)		2 (11)	46 (14)		30 (44)	20 (7)	
Positive	301 (87)	19 (79)		23 (72)	300 (88)		16 (89)	294 (86)		39 (56)	280 (93)	
Missing	11	1		0	12		1	10		2	9	
PR			1.00^b^			0.11^b^			1.00^a^			< 0.001^b^
Negative	79 (24)	6 (25)		11 (38)	75 (23)		4 (25)	77 (24)		33 (53)	53 (19)	
Positive	246 (76)	18 (75)		18 (62)	248 (77)		12 (75)	244 (76)		29 (47)	233 (81)	
Missing	32	1		3	30		3	29		9	23	
HER2			1.00^a^			0.10^a^			1.00^a^			0.12^b^
Negative	295 (86)	21 (88)		24 (75)	295 (87)		16 (89)	292 (86)		63 (93)	253 (85)	
Positive	49 (14)	3 (12)		8 (25)	44 (13)		2 (11)	48 (14)		5 (7)	46 (15)	
Missing	13	1		0	14		1	10				
Ki67			0.19^b^			0.004^b^			1.00^b^			< 0.001^b^
Low	216 (63)	11 (48)		12 (38)	217 (64)		11 (61)	210 (62)		25 (37)	201 (67)	
High	129 (37)	12 (52)		20 (62)	122 (36)		7 (39)	128 (38)		43 (63)	97 (33)	
Missing	12	2		0	14		1	12		3	11	
St Gallen subtype												
Luminal A	122 (37)	9 (39)	0.27^a^	7 (23)	126 (39)	0.02^a^	6 (40)	123 (38)	1.00^a^	11 (18)	120 (42)	< 0.001^b^
LuminalB/HER2−	108 (33)	6 (26)		7 (23)	108 (33)		5 (33)	104 (32)		19 (31)	94 (33)	
Luminal B/HER2+	63 (19)	3 (13)		9 (29)	57 (18)		3 (20)	59 (18)		6 (10)	59 (20)	
HER2+	11 (3)	0 (0)		1 (3)	10 (3)		0 (0)	11 (3)		3 (5)	8 (3)	
Triple-negative	26 (8)	5 (22)		7 (23)	24 (7)		1 (7)	29 (9)		23 (37)	8 (3)	
Missing	27	2		1	28		4	24		9	20	
N-cadherin			0.05^a^									
Negative	330 (92)	20 (80)										
Positive	27 (8)	5 (20)										
Twist			0.11^a^			0.18^a^						
Negative	326 (95)	20 (87)		26 (90)	323 (95)							
Positive	16 (5)	3 (13)		3 (10)	16 (5)							
Missing	15	2		3	14							
Vimentin			0.01^a^			< 0.001^b^			0.36^a^			
Negative	291 (83)	13 (56)		17 (55)	290 (84)		14 (74)	288 (82)				
Positive	61 (17)	10 (44)		14 (45)	57 (16)		5 (26)	62 (18)				
Missing	5	2		1	6		0	0				

*ER* estrogen receptor, *HER2* human epidermal growth factor receptor 2, *N* number, *NHG* Nottingham histological grade, *PR* progesterone receptor

^a^*P* value from Fisher's exact test

^b^*P* value from Pearsons *χ*^2^ test

### Patient and tumor characteristics in relation to primary tumor expression of EMT-related markers and phenotype

**Fig. 2:**
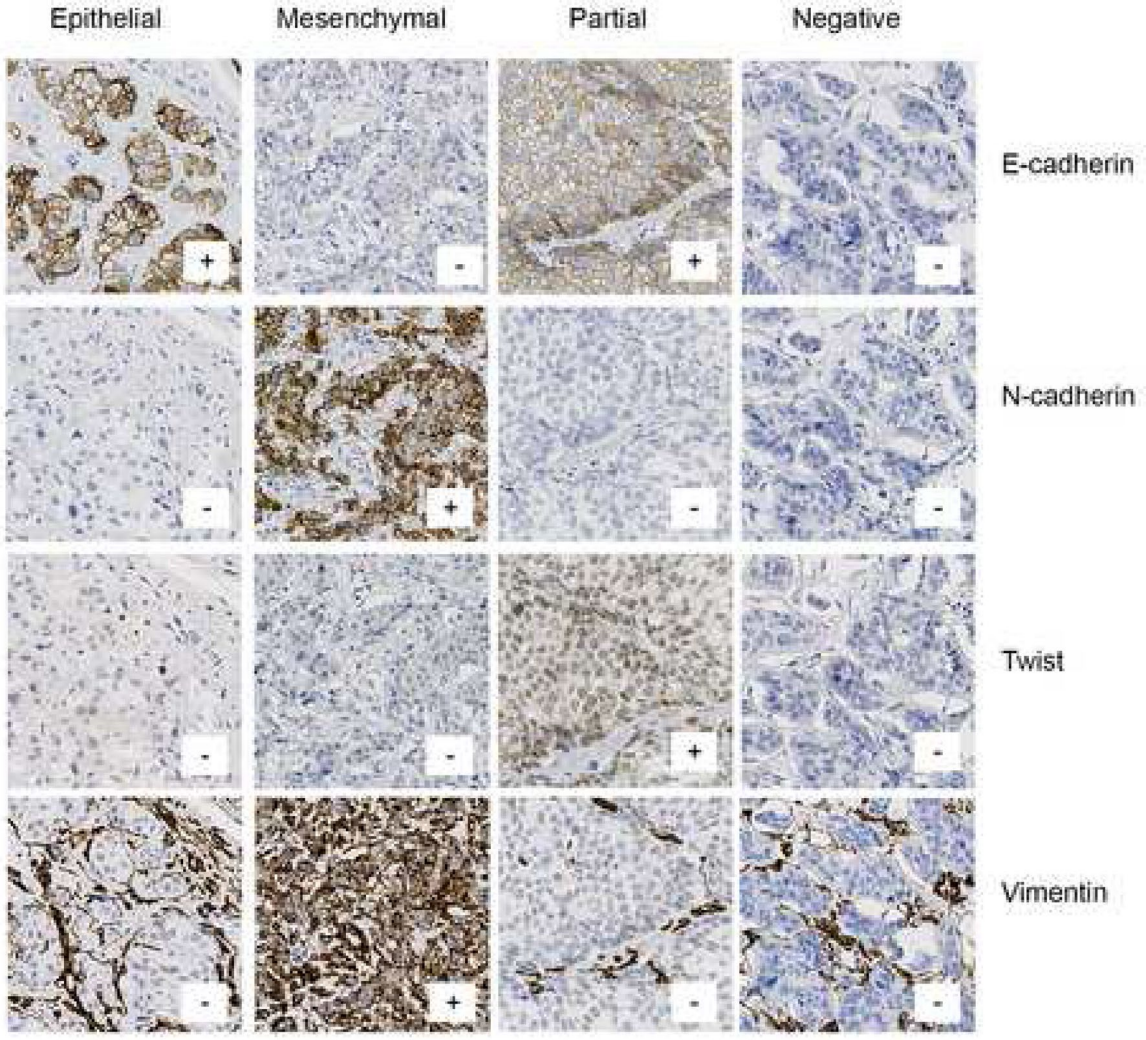
Representative cases of immunohistochemistry stainings for E-cadherin, N-cadherin, twist and vimentin, and of each epithelial-mesenchymal transition (EMT) phenotype. All samples evaluable for E-cadherin (epithelial marker) and at least one mesenchymal marker (N-cadherin, twist, vimentin) were stratified into to the following phenotypes of EMT: epithelial type (positive expression of epithelial marker and negative expression of all evaluable mesenchymal markers); mesenchymal type (negative expression of epithelial marker and positive expression of at least one mesenchymal marker); partial EMT type (positive expression of epithelial marker and of at least one mesenchymal marker); and negative type (negative expression for epithelial marker and all evaluable mesenchymal markers). Original magnification ×40

IHC analysis was successful in 76–92% of cases (Fig. 1). Positive staining of E-cadherin and N-cadherin were localized to the cellular membrane, twist in the nucleus, and vimentin in the cytoplasm. Photomicrographs demonstrating examples of IHC staining are presented in Fig. 2.

Table 1 presents an overview of patient and PT characteristics in relation to PT expression of the EMT-related markers. PT status of E-cadherin and twist were not associated with any clinicopathological factor. Notably, suggestive evidence of an association was seen between N-cadherin positive status in the PT and negative ER status (*P* = 0.03), and triple-negative St Gallen subtype (*P* = 0.02), as well as between vimentin positive status in the PT and younger age (*P* = 0.006), and tumor size > 20 mm (*P* = 0.02). Associations were seen between N-cadherin positive status in the PT and NHG III (*P* < 0.001) and high Ki67 (*P* = 0.004), as well as between vimentin positive status in the PT and NHG III, negative hormone receptor status, high Ki67, and triple-negative St Gallen subtype (*P* < 0.001). PT status of E-cadherin was inversely associated with PT status of vimentin (*P* = 0.01), whereas PT status of N-cadherin was positively associated with PT status of vimentin (*P* < 0.001).

Mesenchymal and partial EMT phenotypes were associated with NHG III, hormone receptor negative status, high Ki67, triple-negative St Gallen subtype as well as epidermal growth factor (EGFR) positivity and platelet derived growth factor C (PDGFC) positivity, whereas epithelial and negative EMT phenotypes were associated with NHG I and II, hormone receptor positive status, low Ki67, and luminal A St Gallen subtype (*P* < 0.001) (Table 2).

**Table 2:** Associations between primary tumor epithelial-mesenchymal transition (EMT) phenotype and patient and primary tumor characteristics in 382 patients with invasive breast cancer

Characteristics	Epithelial, *N* (%)	Mesenchymal, *N* (%)	Partial, *N* (%)	Negative, *N* (%)	*P*
	268	13	89	12	
Age					
< 50	52 (19)	2 (15)	27 (30)	0 (0)	0.04^a^
≥ 50	216 (81)	11 (85)	62 (70)	12 (100)	
Tumor size					
≤ 20 mm	188 (70)	7 (54)	53 (60)	10 (83)	0.12^a^
> 20 mm	80 (30)	6 (46)	36 (40)	2 (17)	
Nodal status					
Negative	148 (57)	8 (67)	51 (58)	8 (67)	0.83^b^
Positive	112 (43)	4 (33)	37 (42)	4 (33)	
Missing	8	1	1	0	
NHG					
I	76 (29)	2 (15)	13 (15)	1 (8)	< 0.001^a^
II	123 (46)	3 (23)	30 (34)	11 (92)	
III	68 (25)	8 (62)	45 (51)	0 (0)	
Missing	1	0	1	0	
ER					
Negative	17 (7)	5 (38)	28 (33)	0 (0)	< 0.001^a^
Positive	243 (93)	8 (62)	58 (67)	11 (100)	
Missing	8	0	3	1	
PR					
Negative	47 (19)	6 (46)	32 (42)	0 (0)	< 0.001^a^
Positive	201 (81)	7 (54)	45 (58)	11 (100)	
Missing	20	0	12	1	
HER2					
Negative	223 (86)	11 (85)	72 (85)	10 (91)	0.94^a^
Positive	36 (14)	2 (15)	13 (15)	1 (9)	
Missing	9	0	4	1	
Ki67					
Low	180 (69)	4 (31)	36 (42)	7 (70)	< 0.001^a^
High	80 (31)	9 (69)	49 (58)	3 (30)	
Missing	8	0	4	2	
St Gallen subtype					
Luminal A	105 (41)	2 (15)	17 (22)	7 (70)	< 0.001^b^
LuminalB/HER2−	84 (33)	4 (31)	24 (32)	2 (20)	
Luminal B/HER2+	50 (20)	2 (15)	13 (17)	1 (10)	
HER2+	7 (3)	0 (0)	4 (5)	0 (0)	
Triple-negative	8 (3)	5 (39)	18 (24)	0 (0)	
Missing	14	0	13	2	
EGFR					
Negative	229 (88)	9 (69)	50 (59)	10 (91)	< 0.001^a^
Positive	32 (12)	4 (31)	35 (41)	1 (9)	
Missing	7	0	4	1	
PDGFC					
Negative	222 (85)	7 (54)	42 (51)	10 (83)	< 0.001^a^
Positive	38 (15)	6 (46)	41 (49)	2 (17)	
Missing	8	0	6	0	

*ER* estrogen receptor, *EGFR* epidermal growth factor receptor, *HER2* human epidermal growth factor receptor 2, *N* number, *NHG* Nottingham histological grade, *PDGFC* platelet derived growth factor C, *PR* progesterone receptor

^a^*P* value from Fisher's exact test

^b^*P* value from Pearsons *χ*^2^ test

### EMT-related marker status across tumor progression stages

E-cadherin expression was downregulated more frequently in recurrences, whereas vimentin was more frequently expressed in recurrences, when compared to expression in the PTs and LNMs. No recurrence was positive regarding the expression of twist (Table 3).

**Table 3:** Distribution of epithelial-mesenchymal transition (EMT)-related marker and phenotype status in the subsets of patients included at different stages

	Primary tumor	Synchronous LNM	Recurrence
	(*N* = 419)	(*N* = 131)	(*N* = 34)
	*N* (%)	*N* (%)	*N* (%)
EMT-related marker status			
E-cadherin			
Positive	357 (93)	105 (95)	22 (81)
Negative	25 (7)	6 (5)	5 (19)
Missing	37	20	7
N-cadherin			
Positive	32 (8)	11 (10)	1 (4)
Negative	353 (92)	99 (90)	26 (96)
Missing	34	20	7
Twist			
Positive	19 (5)	6 (6)	0 (0)
Negative	350 (95)	100 (94)	26 (100)
Missing	50	25	8
Vimentin			
Positive	71 (19)	13 (12)	8 (31)
Negative	309 (81)	93 (88)	18 (69)
Missing	39	25	8
EMT phenotype			
Epithelial	268 (70)	82 (74)	13 (48)
Mesenchymal	13 (4)	2 (2)	0 (0)
Partial	89 (23)	23 (21)	9 (33)
Negative	12 (3)	3 (3)	5 (19)
Missing	37	21	7

*EMT* epithelial-mesenchymal transition, *N* number, *LNM* lymph node metastasis

The expression of each EMT-related marker was compared across the different tumor progression sites as paired data (Table 4). A total of 103 cases had paired data regarding E-cadherin expression between PTs and synchronous LNMs and a discordance rate of 5% was observed. An E-cadherin discordance rate of 17% was observed between 23 paired PTs and recurrences. Regarding N-cadherin expression discordance rates of 9% and 13% were observed between PTs and synchronous LNMs (103 pairs), and PTs and recurrences (24 pairs), respectively. Twist expression was seen to be more stable between the 92 pairs of PTs and synchronous LNMs, with a discordance rate of 2% only. None of the 20 matched pair samples of PTs and recurrences were twist positive. Discordance rates of vimentin expression were observed in 9% of 97 pairs of PTs and synchronous LNMs, and in 14% of 22 pairs of PTs and recurrences. However, none of the shifts observed were statistically skewed (Exact McNemar test, *P* > 0.05).

**Table 4:** Epithelial-mesenchymal transition (EMT)-related marker and EMT phenotype conversion rate between paired primary tumors and corresponding metastases

EMT- related marker	*N*	Positive in PT	Positive in LNM	Conversion rate PT → LNM			
		*N* (%)	*N* (%)	(−) → ( +) *N* (%)	( +) → (−) *N* (%)	*N* (%)	P*
E-cadherin	103	100 (97)	97 (94)	1 (1)	4 (4)	5 (5)	0.38
N-cadherin	103	7 (7)	10 (10)	6 (6)	3 (3)	9 (9)	0.51
Twist	92	4 (4)	6 (7)	2 (2)	0	2 (2)	0.50
Vimentin	97	18 (19)	13 (13)	2 (2)	7 (7)	9 (9)	0.18

EMT-related marker	*N*	Positive in PT	Positive in REC	Conversion rate PT → REC			
		*N* (%)	*N* (%)	(−) → ( +) *N* (%)	( +) → (−) *N* (%)	*N* (%)	P*
E-cadherin	23	22 (96)	18 (78)	0	4 (17)	4 (17)	0.13
N-cadherin	24	2 (8)	1 (4)	1 (4)	2 (8)	3 (13)	1.00
Twist	20	0	0				
Vimentin	22	6 (27)	7 (32)	2 (9)	1 (5)	3 (14)	1.00

EMT phenotype	*N*	EMT phenotype in PT	EMT phenotype in LNM	Conversion rate PT → LNM			
		*N* (%)	*N* (%)	(−) → ( +) *N* (%)	( +) → (−) *N* (%)	*N* (%)	P*
Epithelial (vs all other)	102	77 (76)	75 (74)	8 (8)	10 (10)	18 (18)	0.82
Mesenchymal (vs all other)	102	2 (2)	2 (2)	1 (1)	1 (1)	2 (2)	1.00
Partial (vs all other)	102	22 (22)	22 (22)	9 (9)	9 (9)	18 (18)	1.00
Negative (vs all other)	102	1 (1)	3 (3)	2 (2)	0	2 (2)	0.50

EMT phenotype	*N*	EMT phenotype in PT	EMT phenotype in REC	Conversion rate PT → REC			
		*N* (%)	*N* (%)	(−) → ( +) *N* (%)	( +) → (−) *N* (%)	*N* (%)	*P* *
Epithelial (vs all other)	23	15 (65)	9 (39)	1 (4)	7 (30)	8 (35)	0.07
Mesenchymal (vs all other)	23	0	0				
Partial (vs all other)	23	7 (30)	9 (39)	4 (17)	2 (9)	6 (26)	0.69
Negative (vs all other)	23	1 (4)	5 (22)	4 (17)	0	4 (17)	0.13

*EMT* epithelial-mesenchymal transition, *N* number, *LNM* lymph node metastasis, *PT* primary tumor, *REC*, recurrence

*Exact McNemar test

### EMT phenotypes across tumor progression stages

Based upon combined IHC results for E-cadherin (epithelial marker) and N-cadherin, twist, and vimentin (mesenchymal markers) an EMT phenotype could be assigned to 382 PTs, 110 LNMs and 27 recurrences, of which 102 and 23 had paired data, respectively.

In recurrences the epithelial phenotype was less frequently observed, whereas partial EMT and negative phenotypes were more frequently seen, compared to the phenotype distribution in PT and LNM. No recurrence was classified as mesenchymal phenotype (Table 3).

An overall EMT phenotype conversion was seen in 20% of cases between PT and LNM, and in 39% between PT and recurrence. The evidence for difference in EMT phenotypes between pairs of PT and LNM, or PT and recurrence was generally weak when analyzed by exact McNemar test (Table 4). However, a trend towards a shift from epithelial type in PT to any other phenotype in the recurrence was seen (*P* = 0.07). An EMT phenotype conversion was seen in 8/23 (35%) cases when looking at the epithelial vs non-epithelial phenotype between matched pairs of PT and recurrence samples, 88% (7/8) of which changed from a epithelial to non-epithelial phenotype.

### EMT-related marker and phenotype status and patient outcome

**Fig. 3:**
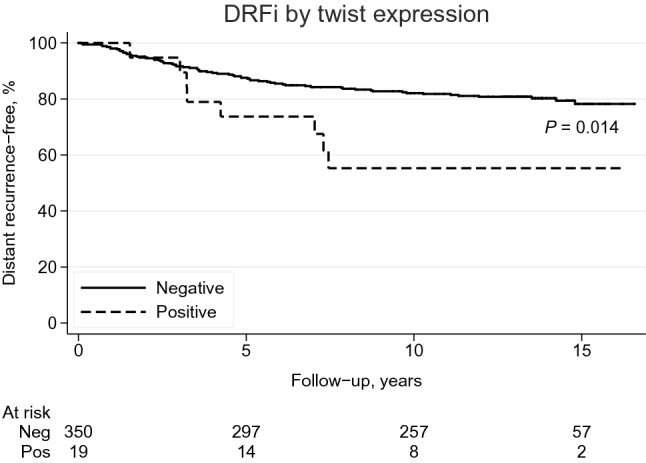
Kaplan–Meier survival curves showing distant recurrence-free interval (DRFi; years) in relation to twist status in primary tumor. *P* value from log rank test

Kaplan–Meier analyses and log rank test revealed that patients with twist positive PTs had shorter DRFi compared to patients with twist negative PTs (*P* = 0.014) (Fig. 3). The Cox univariable regression analysis confirmed that PT status of twist was associated with DRFi [(HR) 2.4, 95% confidence interval (CI) 1.2–5.1, *P* = 0.02], and this result remained essentially the same (HR 2.5, 95% CI 0.97–6.6, *P* = 0.06) in the Cox multivariable analysis (Table 5). Neither PT status of E-cadherin, N-cadherin, vimentin, nor EMT phenotype, or status of E-cadherin, N-cadherin, twist, vimentin or EMT phenotype in LNMs did predict DFRi. When evaluating the overall shift of EMT phenotype by comparing two sites, there was no difference in DRFi between patients with a shift and no shift of phenotype (Supplementary Fig. 1/Online Resource 2).

**Table 5:** Cox univariate and multivariate analysis of distant recurrence-free interval

	*N*	Univariable (*N* ≤ 419)		Multivariable (*N* = 331)	
		HR (CI)	*P*	HR (CI)	*P*
Age					
< 50 years	90	1.00		1.00	
≥ 50 years	329	0.93 (0.56–0.1.5)	0.78	1.4 (0.77–2.6)	0.27
Tumor size					
≤ 20 mm	285	1.00		1.00	
> 20 mm	134	3.0 (2.0–4.6)	< 0.001	2.0 (1.2–3.3)	0.008
Nodal status					
Negative	235	1.00		1.00	
Positive	174	3.9 (2.4–6.2)	< 0.001	3.9 (2.3–6.8)	< 0.001
NHG					
I	108	1.00		1.00	
II	179	2.7 (1.3–5.6)	0.007	2.6 (0.99–6.9)	0.05
III	127	4.5 (2.2–9.3)	< 0.001	2.8 (1.0–7.8)	0.05
St Gallen subtype					
Luminal A	145	1.00		1.00	
Luminal B HER2−	121	2.8 (1.5–5.2)	0.002	2.5 (1.2–5.0)	0.01
Luminal B HER2+	67	3.5 (1.8–6.9)	< 0.001	2.4 (1.1–5.1)	0.03
HER2-type	13	2.5 (0.72–8.7)	0.15	1.2 (0.24–5.5)	0.87
Triple-negative	33	3.9 (1.7–8.7)	0.001	2.8 (1.1–7.4)	0.04
Twist					
Negative	350	1.00		1.00	
Positive	19	2.4 (1.2–5.1)	0.02	2.5 (0.97–6.6)	0.06

*CI* confidence interval, *HER2* human epidermal growth factor receptor 2, *HR* hazard ratio, *N* number, *NHG* Nottingham histological grade

## Discussion

In the present study, we assessed EMT-related proteins in matched samples of PTs, asynchronous LNMs and recurrences to test the hypothesis that EMT profiles are unstable throughout tumor progression in breast cancer patients. We found discordance rates of expression of single EMT-related markers and defined EMT phenotypes between matched tumor samples in the range of 2–35%. Interestingly, non-epithelial phenotypes were more frequently identified in recurrences compared to in PTs and LNMs. However, in paired analysis between tumor progression sites, including only patients with paired marker readings, the evidence was generally low for change in EMT-related markers and EMT phenotypes. This lack of evidence should, however, not be interpreted as no change, as the power to detect change with the sample size in this study is low. PTs with a positive N-cadherin, positive vimentin, mesenchymal or partial EMT status were associated with more aggressive tumor characteristics, exemplified by the triple-negative subtype. We further evaluated the clinical significance of EMT-related markers and EMT phenotypes, and found that twist positive status of the PT was a negative prognostic factor for DRFi.

Previous studies have compared IHC status of the EMT-related markers included in our study between tumor progression sites, however, mostly comparing PTs and LNMs, and only few studies have described conversion rates of status shifts between matched samples [10, 13, 28, 31, 32, 41]. Overall, our study showed a similar expression of the EMT-related markers and phenotypes in PTs and LNMs, as compared to the expression in recurrences. In contrast to what has been reported previously in distant metastases [10], we found that E-cadherin was expressed less frequently in recurrences. Accordingly, loss of epithelial phenotype and gain of partial EMT and negative phenotypes were more frequently observed in recurrences, as compared to the phenotype distribution in PTs and LNMs. Interestingly, none of the recurrence samples were classified as mesenchymal or twist positive, suggesting that these characteristics do not provide the same advantage at the secondary tumor site when the tumor cells need to re-epithelialize, as it might do in the PT.

To the best of our knowledge, only two previous breast cancer studies have evaluated tumor tissue regarding EMT-related markers in combination to evaluate distinct EMT phenotypes [13, 16]. We used a four-marker panel to define the EMT phenotypes and found a similar distribution as reported previously in a large study classifying EMT phenotypes based upon the expression pattern of E-cadherin and fibronectin in PT samples from 1495 breast cancer patients [16]. A smaller study of EMT phenotypes based upon combined expression of E-cadherin and vimentin in 176 breast PT samples reported a lower fraction of the epithelial subtype and consequently a higher fraction of mesenchymal, partial EMT and negative subtypes [13]. Discrepancies of phenotype frequencies might be a consequence of investigating a panel of four compared to two markers only, as well as of excluding all lobular tumors from our study cohort. As in the two previous studies, we were able to confirm the existence of tumor specimen with a partial EMT phenotype in breast cancer, however, the method used in our study makes it impossible to distinguish the coexistence of both epithelial and mesenchymal tumor cells from the presence of true ‘double positive’ tumor cells.

Positivity of N-cadherin and vimentin were seen to be associated with tumor aggressiveness, consistent with previous reports [42, 43]. Nevertheless, in this study, we did not see any difference by marker status in relation to prognosis in terms of DRFi. Expression of twist in PTs has also previously been linked to various clinical parameters [44, 45]. However, we did not find a significant association with any tumor or patient characteristic analyzed in this study. Still, we found that the few patients (5%) with a twist positive PT had a shorter DRFi, compared to patients with a twist negative PT. This is in line with results obtained previously, where positive expression of twist has been associated with poor outcome of breast cancer patients [44, 46], though an association between positive twist expression and a superior overall survival also has been described [33]. Our results might seem paradoxical, though twist is a transcription factor involved in multiple signaling pathways and could be a more biologically relevant marker to study compared to the three EMT effector markers included in our study [47, 48].

Furthermore, in our study, the mesenchymal and partial EMT phenotypes were associated with more aggressive tumor characteristics, such as the triple-negative subtype, in line with what has been reported previously [13, 16]. A novel finding in the present study is that the partial and mesenchymal EMT phenotypes displayed a high fraction of EGFR and PDGFC positivity compared with the epithelial and negative phenotypes, further supporting that these phenotypes define an aggressive type of breast cancer. EGFR is a hall-mark of basal like breast cancer and has repeatedly been presented as a key player promoting EMT [49, 50]. Interestingly, PDGFC is also associated with features of inferior prognosis in human breast cancer [27] and the PDGFC gene strongly correlates with gene-sets defining the EMT pathways supporting an association also for PDGFC with EMT [51]. The functional role of PDGFC in the EMT promoting process is, however, not settled. EMT phenotype has also been seen to provide prognostic information and patients with a partial EMT phenotype to exhibit higher risk of recurrence and inferior survival [13, 16]. We did not find any differences in DRFi according to PT EMT phenotype in our study. Of note, the published studies are evaluating other endpoints than we did, and we excluded patients with lobular cancers, which Bae et al. did not. Importantly our study included only one fourth of the amount of samples included in the Bae et al. [16] study, and thus have a weaker statistical power to perform subgroup analysis.

This study has several strengths, including assessment of EMT-related markers on tissue samples available from 75% of the participants included in the BMM cohort, prospectively defined hypotheses and analysis plan, evaluation of several EMT-related markers on tumor site pairs by two independent assessors blinded to clinical data, as well as relatively long median follow-up (> 10 years). However, our study also have limitations such as evaluation on TMAs. Nevertheless, similar IHC expression of E-cadherin, N-cadherin, twist, and vimentin between margin and center of primary tumor has been reported, which suggests that no change in our results would be obtained from analysis of whole-tissue sections [32, 52]. Moreover, classification of EMT phenotypes is still controversial and although we have selected representative EMT-related markers there are several other known markers related to EMT that would be relevant to evaluate in this context. In addition, the power to detect significant marker changes between PTs and recurrences was low due to limited amount of sample pairs. Furthermore, a main obstacle is to differentiate tumor cells undergoing EMT from stromal fibroblasts by IHC, and thus it is possible that we have underestimated the extent of stained tumor cells, especially considering a marker like vimentin which is present in fibroblasts [53].

In summary, the study confirms the association between single EMT-related markers and specific EMT phenotypes to aggressive features of the primary tumors and the negative prognostic information provided by twist expression in PTs. Epithelial phenotype was indicated to be lost between PTs and recurrences as a reflection of tumor progression. Still, we were not able to demonstrate strong evidence for difference in expressed EMT-related markers and defined EMT phenotypes between primary tumors and synchronous LNMs or recurrences. Our findings may be explained by underlying biology and add to the current knowledge of breast cancer progression, and suggests further investigation in larger cohorts.

## Electronic supplementary material

Below is the link to the electronic supplementary material.

Supplementary file1 (XLSX 12 kb)

Supplementary file2 (PDF 564 kb)

## Data Availability

The datasets generated during and/or analyzed during the current study are available from the corresponding author on reasonable request.
