# Supplementary material for: Expression of epithelial-mesenchymal transition-related markers and phenotypes during breast cancer progression
Source: Breast Cancer Res Treat. 2020 Apr 16;181(2):369–81. doi: 10.1007/s10549-020-05627-0 (PMC7188722; doi:10.1007/s10549-020-05627-0)
Supplement: Supplementary file 2 — Supplementary file2 (PDF 564 kb) [file 10549_2020_5627_MOESM2_ESM.pdf]

Expression of epithelial-mesenchymal transition-related markers and phenotypes during breast cancer progression

Breast Cancer Research and Treatment

Authors: Charlotte Levin Tykjær Jørgensen[1], Carina Forsare[1], Pär-Ola Bendahl[1], Anna-Karin Falck[2], Mårten Fernö[1], Kristina Lövgren[1], Kristina Aaltonen[3], Lisa Rydén[4,5]

Corresponding author: Charlotte Levin Tykjær Jørgensen, PhD

Address: Department of Clinical Sciences, Lund, Division of Oncology and Pathology, Medicon Village, Building 404, Lund University, SE-22381 Lund, Sweden

Email: charlotte.levin_tykjaer_jorgensen@med.lu.se

Supplementary Figure 1 a-k. Kaplan-Meier survival curves showing distant recurrence-free interval (DRFi; years) in relation to epithelial-mesenchymal transition (EMT)-related marker and phenotype status in primary tumor and lymph node metastasis. E-cadherin (a-b), N-cadherin (c-d), twist (e-f), vimentin (g-h), EMT phenotype (i-j), EMT phenotype shift (k). *P* value from log rank test.

Abbreviations: EMT, epithelial-mesenchymal transition; PT, primary tumor; LNM, lymph node metastasis; DRFi, disease recurrence-free interval

PT **LNM**

a)

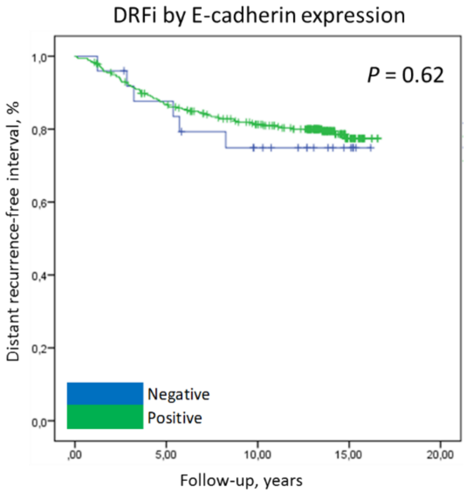

b)

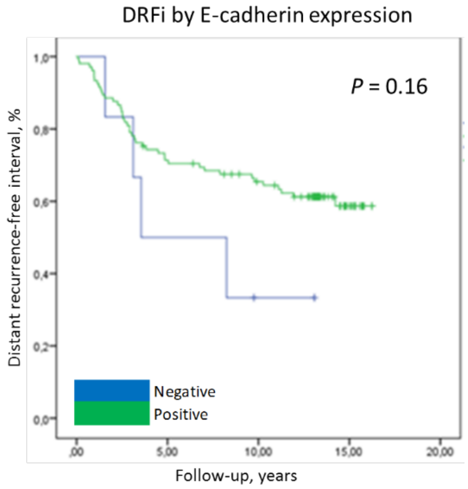

c)

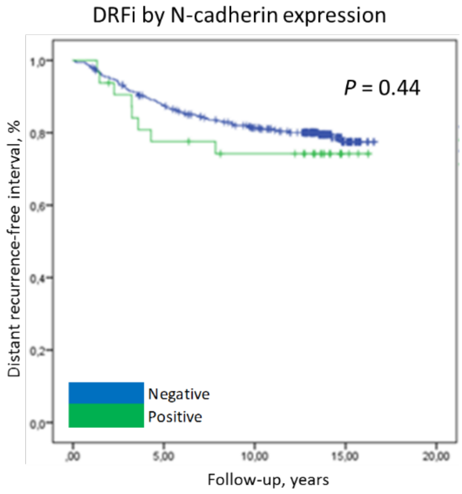

d)

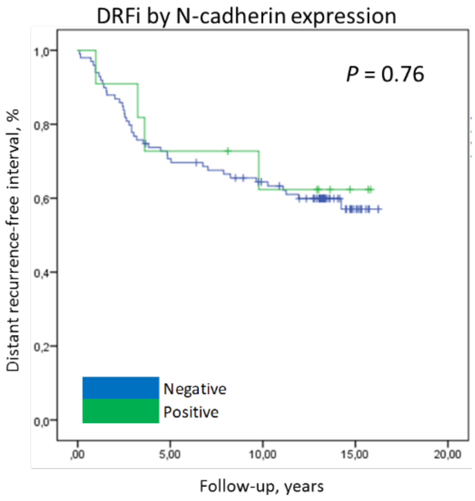

PT | **LNM**

e)

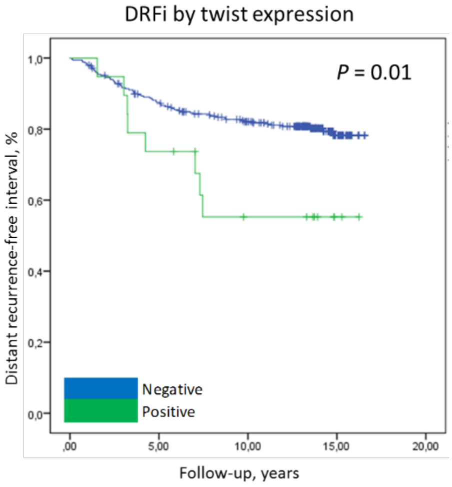

f)

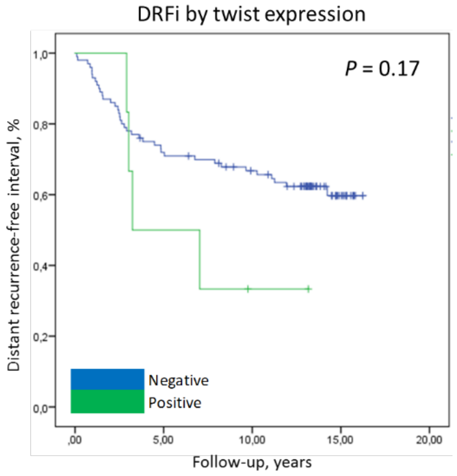

g)

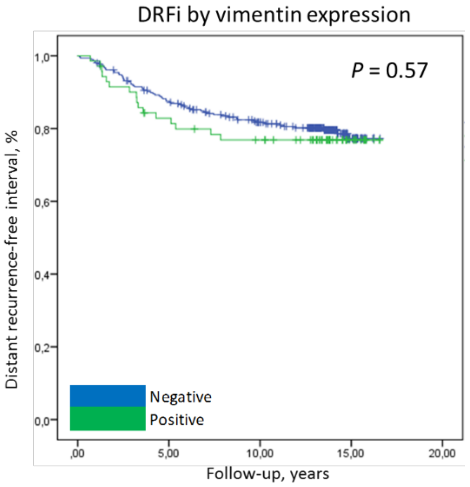

h)

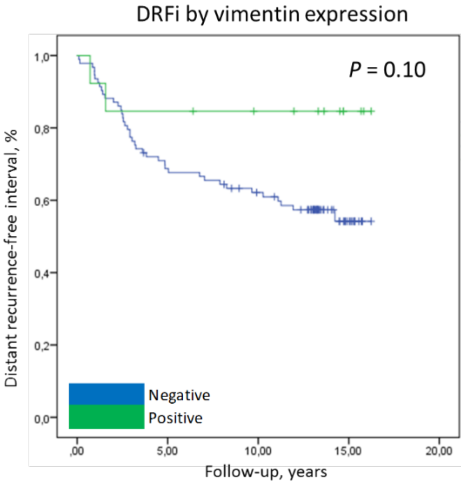

i)

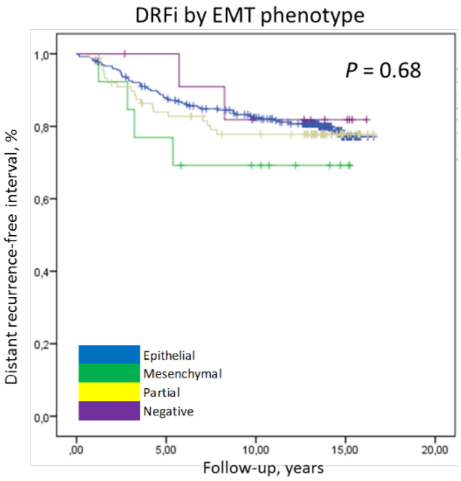

j)

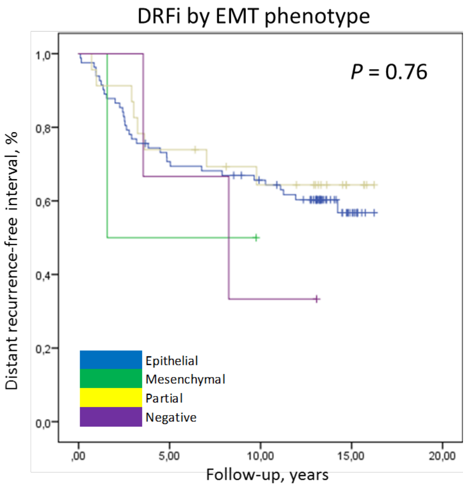

k)

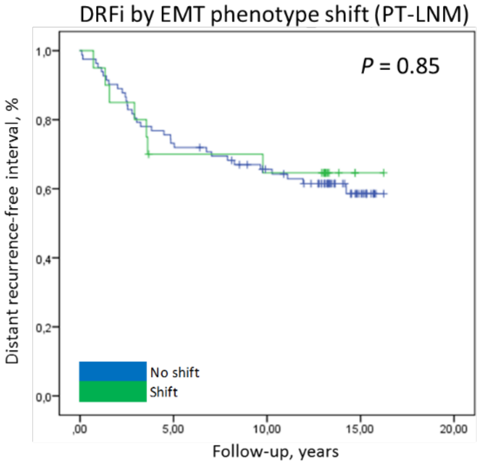
DRFi by EMT phenotype shift (PT-LNM)
P = 0.85
Distant recurrence-free interval, %
1,0
0,8
0,6
0,4
0,2
0,0
No shift
Shift
,00
5,00
10,00
15,00
20,00
Follow-up, years
